# Biomodels

**Published:** 2008-10

**471**

**Antidepressant effect of roots of *Asparagus racemosus* on diabetes - induced depression in rats**

Bhapkar HS^1^, Bansode RL^1^, Jain DP^1^, Singhai AK^2^, Somani RS^1^

**^1^Sinhgad College of Pharmacy, Pune, India; ^2^Dept. of Pharm. Sci.,Dr. HS Gour University, Sagar, India.**

**Objective:**

Depression is twice as common in people with diabetes as in the general population. *Asparagus racemosus* is a widely used Ayurvedic plant, reported for its antidepressant activity. Present investigation aims to study the antidepressant effect of roots of *Asparagus racemosus* on diabetes- induced depression in rats.

**Methods:**

The ethanolic extract of roots of *Asparagus racemosus* (EEAR) was prepared by maceration. Streptozotocin (55mg/kg, i.p.) was given to different groups of rats. Six weeks after injection of STZ, daily EEAR (100 and 200 mg/kg, p.o.) and fluoxetine (5 mg/ kg, i.p.) were administered in diabetic rats (n=5) for one week. The antidepressant activity of EEAR and fluoxetine was studied using Forced swim test (FST), Open field test (OFT), learned helplessness test (LHT) and muricidal behavior. The blood glucose levels were estimated using glucometer.

**Result:**

Six weeks after injection of STZ, diabetic rats exhibited significant depression as compared to normal rats. Daily single dose of EEAR (100 and 200 mg/kg p.o) produced significant (*P*< 0.05) decrease in blood glucose level after six week treatment. The treatment of EEAR and fluoxetine showed significant (*P*< 0.05) decrease in immobility period in FST, increase in the number of squares crossed and rearing episodes and reduced the number of fecal pellets in OFT. The significant (*P*< 0.05) reduction in the number of escape failures concomitant with an increase in avoidance responses in LHT were observed and the incidence of muricide in killer rats reduced.

**Conclusion:**

Thus, *Asparagus racemosus* have potential to decrease diabetes induced depression and needs further studies to elucidate its mode of action.

**472**

**Antidiabetic potential of rhizomes of *Alpinia galanga* in alloxan- induced diabetic rats**

Chudiwal AK^1^, Jain DP^1^, Jain KS^1^, Singhai AK^2^, Somani RS^1^

**^1^Sinhgad College of Pharmacy ^2^Dept. of Pharm. Sci., Dr. HS Gour University, Sagar, India.**

**Objective:**

To study the antidiabetic activity of ethanolic extract of rhizomes of *Alpinia galanga* (EEAG) in normoglycemic and hyperglycemic rats.

**Methods:**

The ethanolic extract of rhizomes of *Alpinia galanga* (EEAG) was prepared by maceration. Single dose of EEAG (50, 100, and 200 mg/kg) was administered orally in normoglycaemic, glucose (1.5 g/kg) fed hyperglycemic and alloxaninduced diabetic rats (n=5). The blood glucose levels were estimated using glucometer. Two weeks treatment of EEAG was carried out in alloxan- induced diabetic rats. The changes in body weight, organ (liver, kidney and pancreas) weight, serum lipid profile and blood parameter (hemoglobin, erythrocytes and differential leukocytes) were assessed after two weeks EEAG treatment. The results of EEAG treatment were compared with diabetic control and normoglycaemic animals.

**Results:**

Single dose administration of EEAG (200 mg/kg) induced a significant (*P*<0.05) decrease in blood glucose level in glucose fed hyperglycemic rats at ½ h compared to control glucose fed rats and in alloxan- induced diabetic rats at 6h after EEAG treatment. After two weeks daily administration of EEAG, blood glucose was significantly (*P*<0.05) reduced as well as diabetic treated rats showed improvement in body weight, organ (liver and pancreas) weight, lipid profiles and hemoglobin content.

**Conclusion:**

Thus, *Alpinia galanga* possesses potential as an antidiabetic and warrants the need for further studies to elucidate its mode of action.

**473**

**Effect of *Tinospora cordifolia* (guduchi) on diabetes induced memory deficit in rats**

Bansode RL^1^, Bhapkar HS^1^, Jain KS^1^, Singhai AK^2^, Somani RS^1^

**^1^Sinhgad College of Pharmacy,Pune, India; ^2^Dept. of Pharm. Sci., Dr. HS Gour University, Sagar, India.**

**Objective:**

Diabetic patients are at increased risk for cognitive decline and dementia. Commonly used Ayurvedic plant, *Tinospora cordiofolia* reported for its role in improving learning and memory. The focus of this study was to find the effect of ethanolic extract of roots of *Tinospora cordifolia* on diabetes induced memory deficit in rats.

**Methods:**

The ethanolic (95%) extract of roots of *Tinospora cordifolia* (EETC) was prepared by maceration. Streptozotocin (55mg/kg, i.p.) was given to different groups of rats. Starting from third day of experiment till the tenth week, EETC (100, 200 mg/kg, p.o.) and metformin (200mg/kg, p.o.) were administered in diabetic rats. Learning and memory was assessed using Elevated plusmaze (EPM), Morris water maze (MWM), Passive avoidance test (PAT) and Brain Acetyl cholinesterase (AchE) levels. Blood glucose levels were estimated using glucometer.

**Result:**

Administration of streptozotocin caused increase in blood glucose and impairment of learning and memory in rats. Daily administration of EETC and metformin produced significant (*P*< 0.05) decrease in blood glucose level after ten weeks. The EETC showed significant decrease in transfer latency in EPM, latency to reach platform was decreased in MWM and increased transfer latency time in PAT. The treatment of EETC significantly reduced brain AchE levels as compared to control diabetic rats.

**Conclusion:**

Thus, *Tinospora cordifolia* possesses potential to attenuate diabetes associated cognitive decline in rats. Further studies are required to find its mode of action in diabetes induced memory deficit.

**474**

**Antinociceptive activity of ethanolic extract of rhizomes of *Alpinia galanga***

Shivgunde PP^1^, Shah P^1^, Jain DP^1^, Singhai AK^2^, Somani RS^1^

**^1^Sinhgad College of Pharmacy,Pune, ^2^Dept. of Pharm. Sci., Dr. HS Gour University, Sagar, India.**

**Objective:**

*Alpinia galanga* is a well-known medicinal plant that has been used in the traditional medicine for treating various diseases including analgesic and anti-inflammatory. Therefore, the aim of present study was to evaluate antinociceptive activity of ethanolic extract of *Alpinia galanga* (Family: Zinziberaceae).

**Methods:**

The ethanolic (95%) extract of rhizomes of *Alpinia galanga* (EEAG) was prepared by maceration. EEAG was examined for analgesic activity using hot plate, formalin induced paw licking and acetic acid induced writhing method. The doses 200 mg/kg and 400 mg/ kg were administered orally.

**Results:**

The results obtained showed that the treatment with EEAG (200 and 400 mg/kg, p.o.) significantly increased pain threshold in hot plate method, produced a marked inhibition of the nociceptive response in both neurogenic and inflammatory phases of formalin test and decreased number of writhings in acetic acid induced nociception.

**Conclusion:**

*Alpinia galanga* showed antinociceptive activity in hot plate, formalin induced paw licking and acetic acid induced writhing method. Hence, it possesses both central as well as peripheral analgesic activity.

**475**

**Short- term calorie restriction decreases oxidative stress in liver mitochondria**

Maheshwari T Doddamane, Verma SK, Singh V, Sharma AR, Singh SN

**Defence Institute of Physiology and Allied Science, Delhi, India.**

Calorie restriction (CR), a dietary regimen with lower food intake without causing malnutrition is being considered to have several benefits including protection from degenerative diseases and delay in aging process. The reduction in oxidative stress is postulated as mechanism of anti-aging effects of CR. It is reported that CR attenuates the age associated increase in mitochondrial super oxide and H_2_O_2_ production, lipid per oxidation, protein oxidation, and oxidative damage to DNA. Effect of 25 and 50% dietary restriction on activity of mitochondrial antioxidant enzymes, protein carbonyl, lipid peroxidation and nitric oxide production was evaluated. Male Sprague Dawley rats were divided in three groups and group 1 and 2 were subjected to 25 and 50% CR (intakes equivalent to 75 and 50% of ad *libitum*) for a period of 15 days and 3^rd^ group without food restriction served as control. The levels of protein carbonyl and lipid peroxidation were found decreased significantly (*P*<0.05) in liver mitochondria of 50% CR rats. The super oxide dismutase (SOD) activity was increased in case of both CR groups (*P*<0.01) in comparison with control. The nitric oxide production was significantly decreased (*P*<0.01) in liver mitochondria of CR group. The weight loss in 25 and 50% CR were 13 and 28% respectively whereas weight (expressed as per kg body mass) of vital organs i.e. heart, kidney, spleen and brain, leg muscles were maintained. The results of this study indicate that short term CR is able to decrease oxidative stress in liver mitochondria.

**476**

**HAPCODESNPs: A novel methodology to understand the crosstalk between population dynamics and protein structural dynamics**

Silla Y, Balasubramanian C, Elayanambi S, Jitender K, Bhupesh T, Shantanu S

**Indian Genome Variation Consortium, Institute of Genomics and Integrative Biology, Delhi, India.**

Single nucleotide polymorphisms (SNPs) are the most common type of genetic variations in humans. In our current study, we have studied the effect of consecutive non synonymous SNPs (nsSNPs) (which we term HAPCODESNPs or hcSNPs) on a disease associated protein whose structure is known in human. Minor allele frequency of hcSNPs was obtained for Transcobalamin II (TCN II), a transporter protein of vitamin B12, as part of the Indian Genome Variation study (IGV). TCN II maintains B12 homoeostasis which has disease implications in stroke, Alzheimer and cardiovascular diseases. We mapped the hcSNPs onto protein structure and through molecular dynamic simulation, analyzed the changes in protein thermodynamic stability and tried to predict the likely changes in molecular function from the structural context. Three hcSNPs with 5 possible combinatorial possibilities, based on population frequency, were utilized for model generation. All models (wild and SNP mapped) were optimized using conjugate gradient algorithm followed by molecular dynamics. All simulations were carried out in explicit water and counter ions in nanosecond scale. To our knowledge, this is the first report linking population genetics with protein structural dynamics with predictions on the effect of such nsSNPs on protein structure. Our preliminary findings indicate that these SNPs, inspite of not co-coordinating ligand binding, either modulate the flexibility of protein which in turn, may have an effect on the ligand interaction.

**477**

**Pharmacological utility of streptozotocin induced rat model of diabetic retinopathy**

Gupta SK, Agarwal Renu, Kumar Binit, Bhutani Dheeraj, Srivastava Sushma, Agrawal SS

**Department of Ocular Pharmacology, Delhi Institute of Pharmaceutical Sciences and Research, Pushp Vihar, New Delhi, India.**

**Aim:**

Pharmacological utility of STZ-induced rat model for evaluating efficacy of experimental drugs for management of diabetic retinopathy.

**Materials and Methods:**

Eight-weeks-old, 12 *Wistar* rats (250-300g) were randomly divided into Group1 (normal) and group2 (diabetic). After baseline blood glucose estimation, rats were injected with vehicle/STZ (45mg/kg) intraperitoneally in groups1and2 respectively and blood glucose estimations repeated weekly. Observations were made for mortality(daily), bodyweight and fundus photography (weekly) till 12wks. After 12wks rats were sacrificed. Superoxide dismutase (SOD), catalase and reduced glutathione (GSH); TNF-alpha and vasoactive-endothelial-growth-factor (VEGF) were estimated in isolated retinas.

**Results and Discussion:**

Group1 showed increase in bodyweight from 246.33±4.96 to 332.83±13.79 grams (*P*<0.0001) but group2 showed decrease from 285.84 ± 25.66 to 264.62 ± 43.82g (*P*<0.08). Blood glucose levels in group1 did not significantly altered during 12wks but in group2 a significant increase was observed from baseline value of 88.22±6.28 to 483.77±89.45mg%. HbA1C values in groups1 and 2 at 12wks post induction were 7.99±2.37 and 23.31±3.57% respectively (*P*<0.0001). Retinal SOD, catalase and GSH levels reduced to 8.50±1.63, 0.05±0.01, 16.49±0.61 in group2 from 18.26±2.50, 0.23±0.05, 25.27±3.12 respectively in group1 (*P*<0.0001 for all). TNF-alpha was reduced in group2 to 3351.71±124.96 from 5144.22±6.36.26 in group1; VEGF was elevated from 9.02±2.75 in group1 to 26.83±3.57 in group2 (*P*<0.0001). Retinal vessel diameter in group2 increased from 50.27±3.90 pixels in group1 to 57.81±3.75 pixels in group 2 (*P*< 0.001).

**Conclusion:**

The model can be utilized effectively to study efficacy of experimental drugs for management of diabetic retinopathy.

**478**

**Antinociceptive and antipyretic activity of *Swertia chirata* in rats**

Dutt HK, Lata S, Kakkar S, Saxena KK, Kumar A, Srivastava VK

LLRM Medical College, Meerut, India

*Swertia chirata (S. chirata)* an indigenous medicine is being used in India since ancient times both in Ayurveda as well as in Unani system of medicine for a variety of ailments. The present study was conducted to evaluate the antinociceptive and antipyretic activity of the water soluble poriton of the alcoholic extract of *S. chirata* in albino rats (either sex weighing 100 to 150g.) after determining LD50 of the extract in albino mice. The antinociceptive activity of S. chirata was evaluated by tail flick and tail clip methods while the antipyretic activity by inducing pyrexia by brewer’s yeast in groups of albino rats (n=6). The animals were fed with the extract in 200 or 400 or 800mg/kg doses. The results were compared with standard drug aspirin. A significant antinociceptive and antipyretic activity was observed which could be attributed to the inhibition of synthesis and/or release of prostaglandins and other chemical mediators, since the results are comparable to aspirin. However, further studies are required to establish its use as analgesic and antipyretic agent.

